# Road-Related Event Detection and Dissemination Through 5G-Based Vehicle-to-Network-to-Everything Communications

**DOI:** 10.3390/s26123928

**Published:** 2026-06-20

**Authors:** Claudia Campolo, Alessandro Confido, Domenico Gioffrè, Antonella Molinaro, Bruno Pizzimenti, Giuseppe Ruggeri, Domenico Mario Zappalà

**Affiliations:** 1 DIIES Department, University Mediterranea of Reggio Calabria, 89122 Reggio Calabria, Italy; cnflsn94s03h224e@studenti.unirc.it (A.C.); gffdnc00a04f112c@studenti.unirc.it (D.G.); antonella.molinaro@unirc.it (A.M.); bruno.pizzimenti@unirc.it (B.P.); giuseppe.ruggeri@unirc.it (G.R.); domenico.zappala@unirc.it (D.M.Z.); 2 Laboratoire des Signaux et Systémes, CentraleSupélec, Université Paris-Saclay, 91190 Gif-sur-Yvette, France

**Keywords:** V2N2X, connected vehicles, 5G, MQTT, inference, machine learning, DENM

## Abstract

Accurate road-event detection and timely alert message dissemination are essential for the safety of connected and automated vehicles. In many scenarios, alert messages must reach not only nearby vehicles but also remote stakeholders, such as traffic management centers, cloud services, and infrastructure operators. This requirement motivates the adoption of cellular-based communication technologies in addition to short-range vehicle-to-everything (V2X) communications for data dissemination. In this work, we investigate vehicle-to-network-to-everything (V2N2X) communications for the dissemination of alert messages generated after the on-board detection of hazardous road events through machine learning (ML) algorithms. Although V2N2X connectivity is well suited for extending data dissemination beyond the local vehicular environment, its capability to guarantee prompt message delivery under strict latency constraints remains an open challenge, particularly when ML inference is integrated into the end-to-end processing pipeline. To address this issue, we develop and experimentally evaluate a proof-of-concept (PoC) platform that combines real-time road-event detection with relevant message dissemination towards both nearby and remote recipients. The proposed framework leverages 5G connectivity and publish/subscribe messaging protocols. The experimental results showcase that dissemination latency is highly influenced by both the adopted type of 5G deployment (private versus commercial networks) and the load conditions at the message broker.

## 1. Introduction

Advanced Driver-Assistance Systems (ADAS) are expected to significantly benefit from Vehicle-to-Everything (V2X) connectivity, which enables vehicles to perceive hazards beyond the limitations of human vision and conventional onboard sensors, thereby improving road safety and traffic efficiency [1].

The dissemination of safety-critical and road-related information traditionally relies on direct Vehicle-to-Vehicle (V2V) and Vehicle-to-Infrastructure (V2I) communications. However, several scenarios require such information to reach entities beyond the immediate vicinity of the vehicles involved, including road infrastructure owners/operators, traffic management centers, navigation service providers, and automotive cloud platforms. Moreover, the deployment of V2V-capable vehicles is expected to be progressive, suggesting that heterogeneous connectivity will shape the near future.

In this context, the Vehicle-to-Network-to-Everything (V2N2X) communication paradigm, promoted by the Fifth Generation Automotive Association (5GAA), represents a promising solution for enabling reliable and efficient data exchange through cellular networks [2]. By exploiting 5G connectivity, V2N2X communications can support low-latency dissemination of road-event notifications to both nearby and remote stakeholders, thus enabling advanced safety and traffic-efficiency services by providing comprehensive and real-time awareness of the traffic environment.

Pioneering theoretical studies have investigated the performance of V2N2X communications through analytical modeling and simulation-based approaches [3,4,5]. For instance, the authors of [5] evaluate the capability of V2N2X communications to support latency-sensitive and safety-critical services under different connectivity configurations. Nevertheless, despite the growing interest in this topic, experimental V2N2X investigations based on real vehicular deployments remain limited.

This work extends the current state-of-the-art by experimentally assessing the capability of 5G-based V2N2X communications to support the dissemination of road-event notifications in realistic operating conditions. Moreover, unlike previous studies, the proposed framework integrates a complete end-to-end processing and communication chain encompassing: *(i)* road-event generation, *(ii)* event detection and classification through on-board cameras and Machine Learning (ML) algorithms, *(iii)* generation and transmission of alert messages, and *(iv)* reception of notifications by nearby and remote entities.

This paper extends our preliminary study presented in [6], where communication performances was evaluated only under limited experimental conditions. The main contributions of this work are summarized as follows:

- We design an end-to-end framework for road-event detection and data dissemination based on commercial off-the-shelf vehicular and Fifth Generation (5G) devices. The dissemination process relies on standardized European Telecommunications Standards Institute (ETSI) Decentralized Environment Notification Messages (DENMs) [7] and on the well- established application-layer Message Queuing Telemetry Transport (MQTT) protocol [8].
- We develop a road-event detection pipeline tightly integrated with DENM generation and transmission procedures. Without loss of generality, we refer to roadwork sign detection as a representative road-event. The ML model is trained using the world’s largest Mapillary Traffic Sign Dataset (MTSD) street-level dataset [9], enhanced through city-specific data augmentation techniques, and implemented using the well-known You Only Look Once (YOLO) [10] single-stage object detection framework.
- We investigate multiple data dissemination scenarios encompassing *(i)* short-range V2X communications, V2N2X communications, and hybrid solutions combining both of them, and *(ii)* different positions and categories of message recipients.
- We implement the proposed framework and experimentally evaluate its performance, through real experiments, in terms of event detection effectiveness and data dissemination timeliness under a broad range of operational conditions.

The remainder of this paper is organized as follows: Section 2 reviews the relevant background and discusses the motivations underlying this study. Section 3 presents the proposed framework, while Section 4 describes its implementation details. Section 5 discusses the experimental evaluation and obtained results. Finally, Section 6 concludes this paper and outlines future research directions.

## 2. Background and Motivations

This section first provides an overview of ETSI DENMs, which are adopted in this work to disseminate road-hazard notifications. Subsequently, existing studies addressing DENM dissemination performance are reviewed. Finally, recent contributions investigating V2N2X communications as an enabling technology for vehicular data dissemination are discussed, highlighting the research gaps addressed by this work.

### 2.1. DENM Messages

ETSI specifies a communication architecture for Intelligent Transport Systems (ITS), encompassing the *Access*, *Networking & Transport*, *Facilities* and *Application* layers. Within this architecture, the DENM is a Facilities-layer message primarily used to notify road users about hazardous events through ITS communication technologies. DENMs convey information regarding events that may negatively affect road safety or traffic conditions.

**Figure 1:**
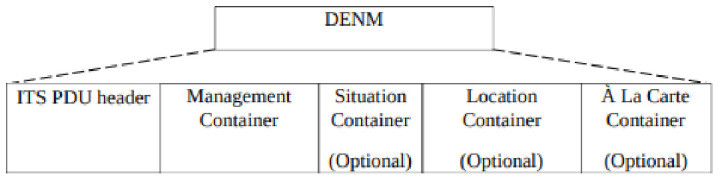
General structure of a DENM.

A DENM consists of a common ITS Protocol Data Unit (PDU) header and a payload structured into multiple containers (Figure 1). According to ETSI specifications [7], the DENM payload includes the following four containers:

- *Management Container*: contains information related to the reliability, evolution, and termination of the detected event; it also enables unambiguous identification of both the originating ITS station and the associated event.
- *Situation Container*: provides information describing the detected event and its potential impact on road safety and traffic flow.
- *Location Container*: includes the geographical position of the event together with its relevance area.
- *À la Carte Container*: carries additional optional information not included in the other containers.

Event classification is specified through the *causeCode* and *subCauseCode* fields contained in the Situation Container. For example, ETSI specifications [7] assign *causeCode* = 2 to Accident with *subCauseCode* = 8 corresponding to assistance requested (e-call); *causeCode* = 3 to Roadworks with *subCauseCode* = 4 representing short-term stationary roadworks; *causeCode* = 94 to Stationary vehicle with *subCauseCode* = 2 indicating vehicle breakdown; *causeCode* = 12 to human presence on the road, whose *subCauseCode* can be used to further extend the classification to specific Vulnerable Road Users (VRUs) such as pedestrians (*subCauseCode* = 4), cyclists (*subCauseCode* = 9), e-scooters (*subCauseCode* = 13) or motorcycles (*subCauseCode* = 19).

Additionally, the *informationQuality* field expresses the confidence level associated with the detected event. Specifically, it represents the probability that the reported event is actually present at the indicated location. The parameter ranges from 0 to 7, where 0 denotes unknown information quality.

### 2.2. Data Dissemination

#### 2.2.1. DENM Delivery

Several simulation-based studies have investigated the dissemination performance of DENMs. Existing works mainly focus on short-range communication technologies, including ITS-G5 [11] and Long Term Evolution (LTE)-V2X/5G-V2X [12], as well as on their co-existence with ETSI Cooperative Awareness Messages (CAMs) [13].

The simulation study presented in [14] analyzes the impact of DENM dissemination on low-priority data traffic, such as infotainment services. The proposed approach introduces a resource allocation scheme designed to prioritize DENM transmissions while exploiting 5G New Radio (NR) V2V broadcast and V2I multicast communications to improve transmission reliability.

The work proposed in [15] addresses large-scale dissemination of DENMs within the road context, through a 5G-enabled V2X infrastructure. In this approach, vehicles detecting hazardous situations broadcast DENMs to nearby V2X gateways deployed along the road infrastructure. The gateways subsequently forward the received information to a traffic management center through HyperText Transfer Protocol (HTTP)-based application programming interfaces (APIs). The traffic management center is then responsible for disseminating notifications to vehicles located outside the proximity of the originating vehicle by exploiting additional gateways. Although the proposed architecture demonstrates the feasibility of extended DENM dissemination, the experimental evaluation highlights significant processing delays that limit its suitability for latency-critical safety message dissemination.

The experimental platform presented in [16] leverages V2X communications to mitigate one of the major limitations of the ITS-G5 technology, namely Non-Line-of-Sight (NLOS) conditions. Specifically, the realized testbed includes a Road Side Unit (RSU) running a YOLO-based Object Detection (OD) algorithm to monitor a safety-critical road segment, such as a sharp bend. Upon detection of an obstacle, the RSU generates and disseminates a DENM to approaching connected vehicles, enabling them to adopt appropriate countermeasures, including emergency braking. The experimental results demonstrate promising performance in terms of both detection accuracy and DENM dissemination latency.

#### 2.2.2. V2N2X Communications

Vehicular communication via the cellular network represents a promising yet not fully explored opportunity for enabling connectivity among heterogeneous V2X stakeholders. In particular, the V2N2X architecture enables interoperable, resilient and scalable information exchange at the application level through a federated cloud-based Information Sharing Domain (ISD), as promoted by the 5GAA [2].

The study reported in [3] provides an analytical model to estimate the latency over the 5G Radio Access Network (RAN). The proposed model considers several radio-related parameters, such as numerology configurations, slot structures, retransmission mechanisms, modulation and coding schemes. This framework enables the identification of suitable radio configurations according to the desired requirements of V2X services under varying distribution and density of vehicles and data traffic features.

In [5], the focus is on the capability of V2N2X communications to support latency-sensitive and safety-critical services under different connectivity configurations for remote recipients. The study evaluates end-to-end latency contributions beyond the 5G RAN, including experimental assessments based on the MQTT publish-subscribe protocol for efficient and scalable data exchange within the ISD architecture.

Low-latency V2N2X communications are further explored in [17], where the authors propose a user-plane packet-routing optimization strategy. In detail, the conventional remote User Plane Function (UPF) of the 5G core network is replaced with an equivalent functionality deployed at the network edge. By reducing the physical distance between the involved User Equipments (UEs) and the routing function, the proposed solution significantly decreases UE-to-network-to-UE latency, as demonstrated through experimental on-field trials conducted over a commercial 5G network.

Our preliminary work presented in [6] experimentally investigates data dissemination latency among vehicles not equipped with direct V2V communication capabilities, but connected through 5G networks. The proposed framework considers a vehicle operating within a private 5G network that detects a hazardous road situation and then generates an MQTT message containing the main DENM fields. Different dissemination scenarios are analyzed, including the case where MQTT publishers and subscribers are hosted on-board vehicles and the MQTT broker is deployed at the network edge. The experimental results highlight the capability of the private 5G network to support dissemination of safety-critical notifications among non-V2V-capable entities within 100 ms, even under broker-loaded conditions.

### 2.3. Contributions

The literature review highlights the lack of a comprehensive experimental investigation of 5G-enabled V2N2X dissemination of road hazard notifications that also includes the event detection stage. Indeed, ML-based inference can introduce a non-negligible latency contribution that affects the overall end-to-end dissemination process.

Existing works addressing inference in the loop, such as [16], focus on short-range communications and assume that hazard detection is performed by an RSU which notifies vehicles.

Instead, studies experimentally investigating 5G-based V2N2X dissemination, including [6,15], do not implement the event detection pipeline and do not consider its impact on the overall dissemination latency.

This work addresses these limitations by providing an integrated experimental framework that combines real-time road-event detection with timely dissemination of DENM notifications through 5G-assisted V2N2X communications. Furthermore, the proposed implementation demonstrates the feasibility of achieving effective event detection and low-latency dissemination using commercially available devices and standardized communication technologies.

## 3. The Proposed Framework

The proposed framework targets the detection of hazardous road events and the dissemination of the corresponding alert information toward intended recipients.

The considered scenario involves a vehicular On Board Unit (OBU) equipped with a camera, embedded computing resources, and ML capabilities able to detect and classify hazardous events from the captured video stream. Upon event detection, the system generates an alert message, carrying the output of the detection, enriched with superimposed geolocation information. The message is disseminated through short-range V2V communications, when available, or through 5G connectivity. The generated notification may target both nearby vehicles and other entities deployed at the network edge.

The following subsections describe the main building blocks of the proposed framework.

### 3.1. Road Event Detection Pipeline

Road events are detected through the analysis of video frames acquired by the onboard camera and processed locally on the vehicular OBU.

Without loss of generality, the following discussion focuses on the detection of roadwork signs. However, the proposed framework and the related findings can be straightforwardly extended to other categories of hazardous road events that can be advertised through DENM, e.g., road accidents, adverse weather conditions, vehicle breakdowns and human or emergency vehicles’ presence on the road.

To satisfy real-time processing constraints, a single-stage, anchor-free object detector is deployed. The input video stream, acquired at a resolution of (1280 × 720) pixels, is letterboxed to the network input size (e.g., 768 × 768), normalized, and forwarded at approximately 20–30 Frame per Second (FPS), depending on platform load conditions. For each candidate location, the model outputs a 4-tuple for the bounding box representation with class logits; when available, an objectness logit [18] is also produced by the export. The post-processing pipeline includes the following stages:

**Score computation:** Let

$$p_{i}\left(\mathrm{cls}\right)=\max_{c}\;\mathrm{softmax}{\left(z_{i}\right)}_{c},\;p_{i}\left(\mathrm{obj}\right)=\sigma \left(o_{i}\right),$$

denote the top 1 class probability (the maximum class probability among all classes for a given detection) and the objectness probability (if present) associated with the *i*-th detection, respectively. The detection score, $s_{i}$, is computed as follows:

$$s_{i}=\left\{\begin{array}{cc} p_{i}\left(\mathrm{obj}\right)\cdot p_{i}\left(\mathrm{cls}\right), & \mathrm{if}\;\mathrm{objectness}\;\mathrm{is}\;\mathrm{considered}\;\mathrm{reliable},\\ p_{i}\left(\mathrm{cls}\right), & \mathrm{otherwise}. \end{array}\right.$$

The reliability of the objectness estimate is evaluated online from the distribution of $p_{i}\left(\mathrm{obj}\right)$ within each frame.

**Class-wise thresholds**: A base confidence threshold τ (the minimum score required for a detection), typically ranging between 0.25 and 0.35, is adapted on a per-class basis to obtain class-specific thresholds ($\tau_{c}$). The threshold values are empirically tuned on the validation set by analyzing the precision–recall trade-off under different operating points. The selected configuration maintains stable precision performance while preserving high recall for safety-critical categories (e.g., *roadworks*, *other-danger*). Class-specific thresholds are introduced to prioritize selected hazard classes by relaxing or tightening the decision boundary according to their safety relevance. Additionally, optional class-specific weights ($w_{c}$) are introduced to amplify scores for prioritized hazards. The weighted score becomes:

$$s_{i}^{\prime }=w_{c(i)}\cdot s_{i}.$$

**Non-Maximum Suppression (NMS)**: Class-wise NMS, formulated as a Local Maximum Search procedure, is applied using an Intersection over Union (IoU) threshold θ∈[0.35,0.50] to remove redundant overlapping detections [19]. When the inference engine exposes an EfficientNMS head, its outputs are directly exploited; otherwise, a lightweight Central Processing Unit (CPU)-based NMS is executed during post-processing.

The framework additionally supports class-specific overrides, including IoU thresholds $\theta_{c}$ and per-class top-*K* limits, to control suppression and retain only the most relevant candidate boxes.

**Temporal consensus**: To mitigate spurious single-frame activations, a hazard hypothesis *h* is validated only if the same class together with a spatially consistent box appears in at least *m* frames over a sliding window of *n* frames (i.e., *m* = 3, *n* = 5). Spatial consistency is tested by IoU≥γ (e.g., γ = 0.3). The final hypothesis confidence is computed as the running median of $s_{i}^{\prime }$ over the observation window. Different (m,n) configurations were evaluated to assess the trade-off between responsiveness and false-positive suppression. The adopted majority wins setting, m=3 over n=5 consecutive frames, was selected because it substantially reduces isolated single-frame false activations while introducing only a limited temporal delay, compatible with the near real-time requirements of the proposed pipeline and the static nature of roadwork sign. In particular, this setting requires a detection to persist for at least three frames within a short observation window, thus filtering unstable predictions caused by motion blur, illumination variations, or partial occlusions.

**Geospatial binding**: Each validated hazard is bound to the Global Navigation Satellite System (GNSS) position of the ego vehicle (WGS-84) at detection time *t*. If camera extrinsic parameters or range proxies are available, a distance estimate refines the eventPosition (e.g., projecting along the ego heading by a capped range *R*). Otherwise, the ego vehicle position itself is used, while the event relevance distance is set to a class-dependent radius *r* (e.g., *r* = 120m for *roadworks*, *r* = 60m for *obstacle-on-the-road*).

### 3.2. Alert Message Generation

Once a hazardous event is detected, the system generates a DENM. An example of a portion of the generated DENM payload is: *{‘stationId’: 33, ‘causeCode’: 3, ‘subCauseCode’: 4, ‘validityDuration‘: 5000,‘eventPosition’: ‘lat’:381211432, ‘lon’: 156591732, ‘informationQuality’: 4}*. The message notifies a change in traffic conditions occurring at a specific geographical location. In the considered example, the *causeCode* field identifies a roadwork-related event, while the *stationId* uniquely identifies the OBU that detected the event.

The detector confidence score is mapped to the DENM *informationQuality* field through a monotonic discretization rule summarized in Table 1. Consequently, higher-confidence detections correspond to higher information-quality levels. This mapping is implemented at the application layer.

### 3.3. Data Dissemination

Different dissemination strategies have been designed and experimentally evaluated in order to address heterogeneous communication conditions and different categories of recipients. The considered scenarios encompass both short-range and long-range dissemination technologies, as well as recipients located either in proximity of the detecting vehicle or remotely at the network edge.

**Figure 2:**
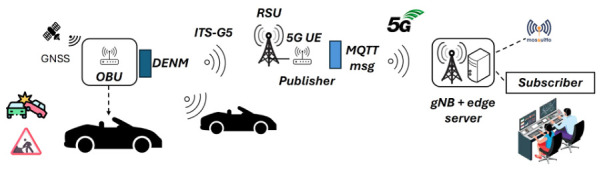
ETSI DENM dissemination to road entities in proximity through ITS-G5 and to entities at the edge through 5G connectivity.

**Figure 3:**
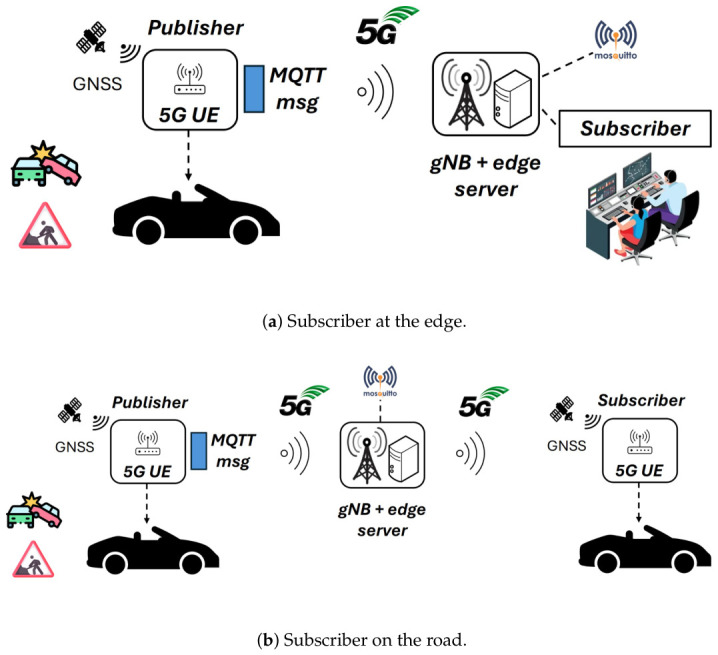
DENM-like message dissemination through 5G connectivity.

In particular, the first scenario (Figure 2) combines short-range ITS-G5 communications with 5G-assisted dissemination, with a detecting vehicle equipped with short-range V2X capabilities only. The remaining scenarios (Figure 3) focus on fully 5G-enabled V2N2X communications.

#### 3.3.1. Hybrid Data Dissemination Scenario

The first scenario, illustrated in Figure 2, considers a vehicle equipped with an ITS-G5-capable OBU that detects a hazardous event through the previously described perception pipeline and subsequently triggers DENM transmission.

The OBU configures the transmission parameters and broadcast DENMs over the ITS-G5 interface. The transmitted DENMs are received by nearby entities, including other OBUs and an RSU.

Upon reception of the DENM, the RSU builds a corresponding JavaScript Object Notation (JSON)-formatted MQTT PUBLISH message and forwards it through the 5G interface. The PUBLISH message is subsequently received by an MQTT broker hosted at the edge of the 5G network which finally forwards it to the intended subscriber at the edge.

Under this data dissemination scenario, intelligent event aggregation at the RSU level can be enforced. Specifically, if multiple vehicles report the same event (e.g., identical roadwork sign at the same location), the RSU can aggregate the received notifications before transmitting the MQTT PUBLISH message which is then received by the broker.

For instance, if multiple DENMs received within a given time window have the same *causeCode* and *subCauseCode*, and their *eventPosition* falls within a given geographical area, the RSU can infer that these DENMs refer to the same detected event. The time window can be reasonably set equal to the *validityDuration* field [7] of the first DENM. Such a DENM field allows us to estimate the duration of an event up to 24 h and can be renewed according to the reception of a novel DENM describing the same event.

However, the definition of the event’s duration and detection area is crucial and depends on the specific type of detected event by the originating vehicle. For instance, some events may last only a few seconds, while others, like roadwork sign, may remain relevant for several hours or days.

Although such aggregation at the RSU can reduce both the traffic over the network and the processing load experienced by the MQTT broker, the specific implementation of the aggregation logic is beyond the scope of the present work and left for future investigation.

#### 3.3.2. 5G-Assisted V2N2X Data Dissemination Scenario

The second scenario considers a vehicle not equipped with direct V2V communication capabilities but connected through a 5G gateway. In this case, the vehicle directly disseminates a DENM-like message through the 5G connectivity, as illustrated in Figure 3. Similarly to the previous scenario, the MQTT broker is hosted at the 5G network edge.

Two dissemination configurations are considered:

- **Subscriber at the edge**: the vehicle publishes a DENM-like message intended to notify of a dangerous road-event entities at the edge (Figure 3a).
- **Subscriber on the road:** the dissemination of DENM-like messages occurs among non-V2V capable vehicles through the 5G network (Figure 3b); in this configuration, one vehicle acts as MQTT publisher while another operates as subscriber.

### 3.4. The Subscriber

The subscriber deployed at the network edge may represent different stakeholders and services, e.g., a road operator, a traffic management application, an emergency dispatch center, and analytics platforms.

In addition, the subscriber may also consist of a Server-Local DynamicMap (S-LDM) [20], a centralized geo-referenced database containing topographical, positional and status-related information characterized by different levels of temporal dynamics. The S-LDM builds upon the concept of Local Dynamic Map (LDM) standardized by ETSI [21]. Transient dynamic information maintained by the LDM can be updated using temporary road conditions disseminated through DENMs, such as speed limits due to roadworks or hazardous road events.

## 4. Implementation

A Proof-of-Concept (PoC) platform bridging together all the conceived architectural building blocks has been developed and experimentally validated. The following subsections describe the main hardware and software elements of the implemented framework.

### 4.1. Vehicle: Main Components

**Figure 4:**
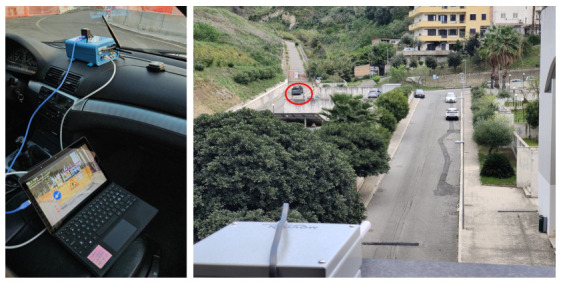
Testbed in the campus premises: OBU mounted on board (**left**) and RSU covering the area where the equipped vehicle (the one within the red circle) moves (**right**).

The test vehicle integrates a front-facing Universal Serial Bus (USB) camera connected to an embedded Graphics Processing Unit (GPU)-based OBU. Figure 4 (left) illustrates both the sensor placement and the in-vehicle architecture.

#### 4.1.1. Camera

The onboard video stream is captured through a See3CAM CU135 USB 3.0 camera featuring a 13 megapixel (MP) sensor, with a nominal 67° field of view, and support for real-time acquisition up to 60 FPS at High Definition (HD) resolution.

#### 4.1.2. Computing Capabilities

The OBU integrates an NVIDIA Jetson AGX Orin module mounted on a Photon carrier board. Such a configuration is getting common in recent off-the-shelf commercial vehicular devices [22,23]. The platform runs Ubuntu 20.04 Long Term Support (LTS) with NVIDIA JetPack 5.1.2 (L4T 35.4.1) over a Linux 5.10 kernel. The hardware platform includes a 12-core Arm Cortex-A78AE v8.2 64-bit CPU clocked up to 2.2 GHz, and configured in MAXN mode, corresponding to the highest performance profile with a power envelope up to 60 W. The system also integrates 64 GB of 256-bit LPDDR5 RAM. GPU-accelerated processing capabilities are provided by an NVIDIA Ampere GPU architecture featuring 2048 CUDA cores, 64 Tensor Cores, and up to 8 GPU Texture Processing Clusters (TPCs) enabled in MAXN mode. The platform is further complemented by 2x NVDLA v2.0 accelerators and a PVA v2.0 engine for vision-oriented workloads.

The NVIDIA software stack is based on CUDA 11.4, cuDNN 8.6, and TensorRT 8.5, enabling high-performance Artificial Intelligence (AI) inference and accelerated computing. Overall, the platform provides up to 5.3 TFLOPS Single-precision Floating Point (FP32) and about 10.6 TFLOPS Half-Precision Floating Point (FP16) of GPU compute performance. FP32 follows the IEEE-754-2019 [24] 32-bit floating point numerical format, commonly used for general-purpose GPU computing, while FP16 refers to the IEEE 754-2008 [25] specification and is widely used for mixed-precision training, AI inference, and tensor-heavy workloads.

#### 4.1.3. V2X Communication Capabilities

From a communication perspective, the OBU is equipped with ITS-G5 connectivity and runs an ETSI-compliant ITS communication stack enabling DENM exchange.

In the second scenario, the OBU is augmented with 5G connectivity through a commercial Teltonika RUTX50 gateway connected via Ethernet. The device runs RutOS [26], which is based on the OpenWrt operating system [27]. We extended the legacy operating system with open-source modules supporting MQTT-based data processing and dissemination functionalities.

### 4.2. RSU

A Movyon Electronics RSU is deployed within the campus premises, as shown in Figure 4 (right). The RSU is equipped with ITS-G5 communication capabilities to interact with vehicles in proximity. Similarly to the OBU, the RSU is augmented with 5G connectivity through a commercial Teltonika RUTX50 device connected via Ethernet.

### 4.3. 5G Network Deployment and Configuration

**Figure 5:**
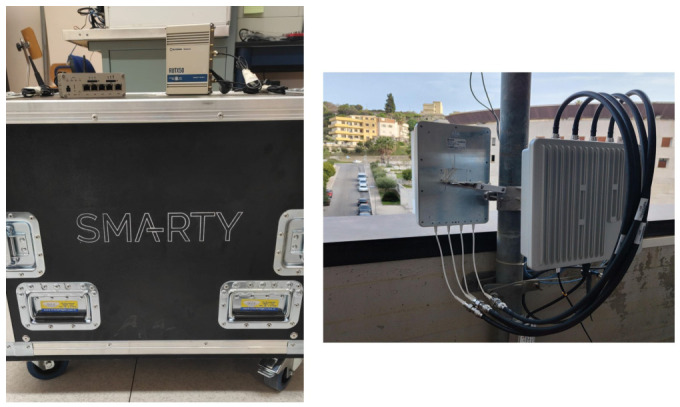
5G private network deployment in the campus: UE, gNB, core network and edge server (**left**) and antenna (**right**).

A private 5G standalone network has been deployed within the campus environment (Figure 5), which operates over licensed spectrum in band n77, centered around 3.99 GHz and allocated for experimental use. The deployment encompasses a complete end-to-end 5G infastructure, including both the RAN and the 5G Core. On the access side, radio coverage is provided by a gNodeB (gNB) serving the experimental area. Access to the private 5G network is achieved through commercial 5G gateways operating as UEs, connected either to the RSU in the first data dissemination scenario, or to the OBU in the second one. Each gateway hosts a Subscriber Identity Module (SIM) provisioned for the private network and containing the subscription credentials required during network registration.

Leveraging a private 5G network allows for performance measurements under controlled RAN conditions that resemble the operation of a dedicated network slice for V2N2X traffic [28].

### 4.4. MQTT Broker Implementation

An edge server co-located with the gNB (Figure 5) runs a containerized instance of the Mosquitto [29] MQTT broker. This edge deployment enables low-latency data dissemination workflows by processing road-event notifications close to the radio access infrastructure. The edge server is equipped with an Intel Core i9-14900KF CPU mounted on a Z690 motherboard, with 64 GB RAM, 1 TB Samsung 990 EVO NVMe storage, and an NVIDIA RTX 4080 SUPER GPU featuring 10,240 CUDA cores and 16 GB GDDR6X memory.

Three different load settings at the broker are considered during the experiments:

- *(i) Light load*: the broker handles one additional client publishing 20 messages per second (∼20 kB/s);
- *(ii) Medium load*: one additional client publishes 500 messages per second (∼5 MB/s);
- *(iii) Heavy load*: the broker handles 8 additional clients transmitting 10 kB payloads in bursts reaching up to 2000 messages/s per client.

### 4.5. ML Algorithm

The event detection module relies on a single-stage YOLO-family object detector trained and exported using the Ultralytics implementation. In particular, the yolo11m model variant is fine-tuned for traffic-sign hazard detection and deployed on the OBU for real-time inference [10].

The trained network is exported to the Open Neural Network Exchange (ONNX) format [30] and then converted into a TensorRT (TRT) engine [31] (TRT is a Software Developmet Kit (SDK) for high-performance deep learning inference on NVIDIA GPUs) for accelerated inference on the embedded Ultralytics platform [32]. More details are reported in the following.

**Dataset and splits**: The fully annotated track of the MTSD [9] is adopted. The original dataset structure consists of three training subsets, one validation subset, and one test subset, corresponding approximately to a 70/10/20 partition for train/validation/test. MTSD is currently one of the largest and most diverse traffic sign datasets available, consisting of over 100,000 high-resolution images captured worldwide under heterogeneous environmental conditions, relating to weather, seasons, and daytime. The dataset provides fine-grained annotations for traffic-sign classes. Models that are trained with this dataset typically exhibit good generalization performance across different road environments.

**City-specific augmentation (Reggio Calabria)**: To mitigate domain-shift effects, we extended the MTSD with a city-specific subset collected in Reggio Calabria. Images were captured under different urban cotexts, times of day, viewpoints, and weather conditions, and manually annotated using the Computer Vision Annotation Tool (CVAT) with bounding boxes for the same taxonomy used in MTSD. Annotations were exported from CVAT into YOLO-compatible labels and ingested into the existing conversion pipeline, while coherently updating data.yaml. The new samples were merged into the training corpus using a stratified policy to preserve per-class balance. A small holdout was reserved for validation to monitor overfitting on local appearance.

During training, the Reggio Calabria subset benefited from the same augmentations (geometric and photometric) as MTSD; no class names were altered, ensuring label consistency. This targeted augmentation improves robustness against local variations in traffic sign style, mounting height, occlusion, and environmental conditions (such as illumination, weather) characteristics of the deployment area.

**Environment**: All experiments were conducted within a dedicated Python virtual environment (venv) on a workstation to ensure reproducibility of software dependencies and toolchain versions. Training and validation activities were performed on a workstation equipped with an Intel Core i9-14900K CPU, 64 GB RAM, and an NVIDIA RTX 4500 Ada Generation GPU. The system provides 4.1 TB of storage capacity and runs Ubuntu 22.04.5 LTS.

**Annotation conversion and dataset YAML**: The original MTSD JSON annotations were converted into the YOLO annotation format using normalized bounding boxes. In parallel, a coherent dataset descriptor (data.yaml) was generated to reference images and labels associated with the training, validation, and test splits. A fixed and reproducible class list was produced, while optionally ambiguous instances may be skipped. The final dataset layout consists of parallel images/ and labels/ trees for training, validation, and test subsets.

**Training**: A modern single-stage YOLO detector was trained using a 768-pixel input resolution. The schedule uses cosine learning-rate decay with early-stopping patience, and standard spatial and photometric augmentations (mosaic, random erasing, mild rotations, translations, scale, shear, and horizontal flips). Training proceeds on the workstation GPU, saving periodic checkpoints and the best-performing weights according to validation mean Average Precision (mAP).

**Validation**: The trained model was evaluated with the same input size and dataset descriptor used for training. Performance was assessed in terms of mAP. AP is computed as the area under the precision-recall curve for each class, considering detections as true positives when the IoU with the ground-truth box exceeds a predefined threshold [33]. The final mAP metric is obtained by averaging AP over all classes. In addition to ${\mathrm{mAP}}_{50}$ (IoU threshold fixed at 0.50), the Microsoft Common Objects in Context (COCO)-style metric ${\mathrm{mAP}}_{50:95}$ is also reported, defined as the mAP averaged over IoU thresholds ranging from 0.50 to 0.95 with a 0.05 step [34].

**Export without NMS**: For downstream, class-wise post-processing on the edge device, the network is exported to ONNX without built-in NMS. This preserves raw logits and box predictions, allowing for customized score computation, per-class thresholds, and tailored NMS settings aligned with safety requirements.

**TensorRT compilation**: The ONNX model is compiled to a TRT FP16 engine with an optimization profile that balances latency and memory footprint. A timing cache and profiling are employed to speed up subsequent builds and to verify kernel selection.

**Figure 6:**
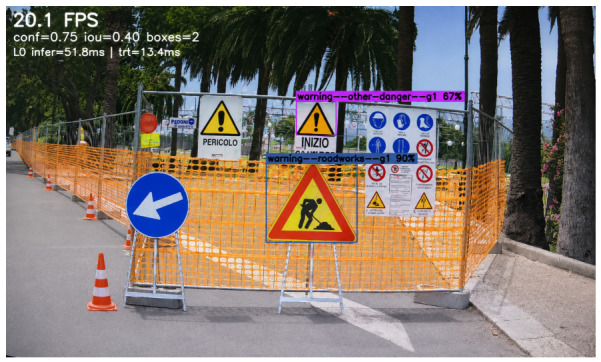
Live inference stream.

**Edge setup and inference**: On the Jetson platform, the live video stream (Figure 6) is processed in real time through the TRT-based inference engine. The application provides both a Motion Joint Photographic Experts Group (MJPEG) preview and a JSON endpoint containing per-frame detections (bounding boxes, predicted classes, and confidence scores) for machine-to-machine consumption.

**Server and V2X bridge**: A Flask-based HTTP server [35] runs on the OBU to expose the TRT detector outputs for both monitoring and downstream V2X processing. The GET / endpoint provides an MJPEG stream with overlaid detections, while GET /detections endpoint returns the latest inference results in JSON format (timestamps, inference latency, and per-object class/score/bounding box). A separate V2X bridge polls /detections, applies event-driven triggering with class filtering and anti-duplication logic. In particular, a new DENM transmission is issued only on the rising edge of a valid hazard detection, i.e., when a target class becomes active after not being previously reported. To avoid repeated reports of the same persistent event, the bridge associates each reported hazard with its class, position, and bounding-box consistency, and suppresses additional notifications during a configurable cooldown interval. This mechanism prevents multiple DENMs from being generated for the same roadwork sign, while it remains visible across consecutive frames after the temporal consensus is reached. The validated detection is then mapped to DENM semantics (*causeCode*/*subCauseCode*, *informationQuality*), and finally the resulting event notification is sent to the ETSI ITS stack via HTTP-based APIs, in case of a DENM transmission over ITS-G5 or published through MQTT otherwise.

## 5. Experimental Assessment

This section first evaluates the performance of the developed inference pipeline and subsequently analyzes the data dissemination latency under different communication scenarios and network conditions.

### 5.1. Inference Results

Table 2 reports the detection performance achieved on the validation dataset through the Ultralytics evaluation pipeline. The trained model is deployed on the Jetson platform and executed through a TRT FP16 engine. End-to-end per-frame inference latency (including preprocessing, GPU execution, and post-processing) is measured through the onboard profiling interface.

The average TRT execution time (considering only GPU inference) is equal to 11.8 ms, while the overall average end-to-end inference latency reaches 52.3 ms. The Jetson platform was configured in *MAXN* power mode, which unlocks the maximum CPU and GPU clock frequencies and removes power constraints to maximize computational throughput during benchmarking.

The gap between the pure TRT execution time (11.8 ms) and the overall inference latency (52.3 ms) mainly derives from CPU-bound additional operations required by the perception pipeline outside the GPU inference kernel, including frame acquisition, image resizing and letterboxing, normalization, host-device memory transfers, score computation, class-wise thresholding, NMS, temporal filtering, and Python-level orchestration. Therefore, the 11.8 ms value represents only the optimized GPU execution stage, whereas the 52.3 ms value reflects the full application-level latency experienced by the system before a detection can trigger the DENM generation. These results confirm that, under the considered configuration, the dominant latency contribution arises from the end-to-end perception pipeline (pre-processing and post-processing stages) rather than from the GPU kernel execution itself.

To quantify the contribution of the Reggio Calabria city-specific augmentation, an evaluation was performed by comparing the detector trained only on the original MTSD dataset against the detector trained on MTSD enriched with the local subset. The evaluation was conducted on the same validation protocol, with particular attention to DENM-related classes. The city-specific augmentation improved the robustness of the detector under local deployment conditions, especially in the presence of non-standard viewpoints, partial occlusions, illumination changes, and locally specific sign placements. As reported in Table 2, the augmented model achieves higher precision, recall, and mAP values on DENM-related classes, confirming the positive impact of the local dataset extension.

Overall, the adopted single-stage detector ensures an effective trade-off between detection accuracy and real-time execution performance for safety-oriented vehicular applications.

### 5.2. Data Dissemination Results

#### 5.2.1. Short-Range Dissemination Delay

**Figure 7:**
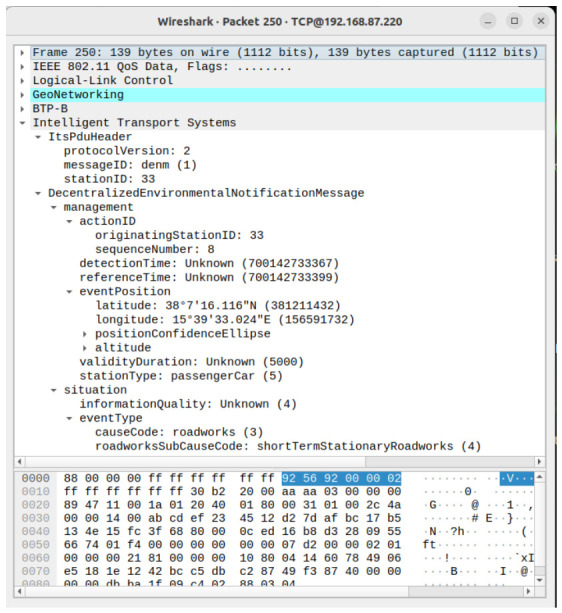
DENM packet captured via Wireshark on the RSU.

In the first experimental scenario, event detection triggers the transmission of a DENM through the ITS-G5 interface. As reported in Figure 7, the transmitted DENM accounts for 139 bytes.

The time passing between event detection and actual DENM transmission over the short-range interface is in the order of 5 ms, mainly due to the usage of HTTP APIs for triggering message generation by the OBU. In particular, the detection-to-API retrieval delay accounts for 4.9 ms, while the HTTP build-and-dispatch latency contributes only 0.1 ms (including approximately 0.03 ms required for payload construction). This shows that the dominant contribution to the overall latency budget is the onboard perception stage.

Once the DENM generation is triggered, transmission over the short-range ITS-G5 interface occurs almost immediately.

#### 5.2.2. 5G-Related Dissemination Delay

**Figure 8:**
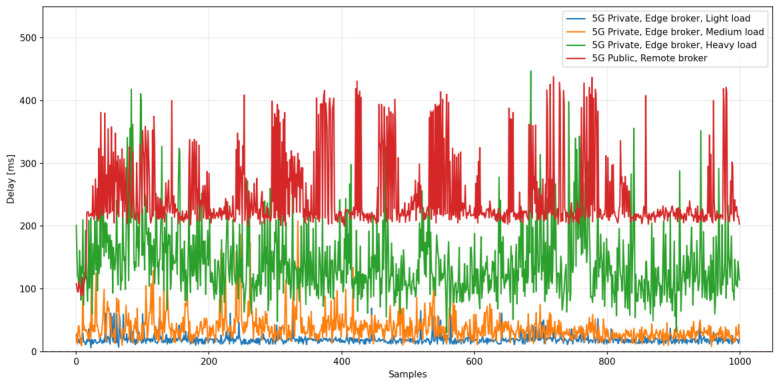
5G-related dissemination delay of 1000 DENMs transmitted under different 5G network and broker load conditions.

The dissemination latency metric reported in Figure 8 measures the time required for a DENM message to be published and sent through the 5G interface and received by a subscriber located at the edge.

In the first dissemination scenario, the metric corresponds to the time elapsed between the RSU publishing the received DENM as an MQTT message and the MQTT subscriber receiving the corresponding MQTT notification.

In the second scenario, the metric measures the dissemination delay of DENM-like notifications at the OBU until the reception at the edge. When dissemination is performed via MQTT, the published DENM has a length of 5958 bytes. Under light broker-load conditions, the average dissemination delay is in the order of 20 ms (blue curve in Figure 8). A moderate increase in the metric is observed when transitioning from light to medium broker load (orange curve), whereas under heavy-load conditions, approximately 2% of samples exceed 300 ms (green curve).

For completeness, the delay experienced for data dissemination through 5G is also measured using a live commercial 5G network together with a remote public *MQTT* broker. The latter is provided by the open-source *EMQX* platform [36], a widely used scalable MQTT 5.0 implementation [37,38,39]. In such a case, both publisher and subscriber are connected through 5G. Although the *EMQX* platform is capable of handling several connections, this setting does not allow us to configure a controlled load on the public broker.

In this configuration, the reduced control over the commercial network and public broker infrastructure results in an average dissemination delay exceeding 200 ms, with approximately 16.8% of samples above 300 ms. More details about the statistical significance of the considered metric are reported in Table 3.

### 5.3. Impact of Duplicate Event Suppression

The anti-duplication mechanism was evaluated to assess its capability to prevent repeated reports of the same road event from unnecessarily increasing the broker load. The mechanism combines a rising-edge trigger with a cooldown interval and spatial consistency checks so that persistent detections of the same roadwork sign generate only one DENM-like notification within the configured temporal window. This behavior is particularly relevant in high-traffic scenarios, where several vehicles may detect and report the same hazardous event.

In the conducted evaluation, repeated detections of the same roadwork sign were processed over continuous video sequences. Without anti-duplication, each valid detection could trigger a new notification, increasing the number of MQTT publications delivered to the broker. With the proposed anti-duplication logic enabled, redundant reports associated with the same event were suppressed, while the first valid notification was preserved.

Table 4 summarizes the obtained results. The proposed mechanism significantly reduces the number of transmitted DENMs, thereby limiting unnecessary broker traffic without preventing the dissemination of safety-relevant information.

## 6. Conclusions and Future Works

This paper investigated the dissemination performance of road-hazard notifications under different communication scenarios while also considering the impact of the event-detection stage within the overall end-to-end dissemination pipeline. The developed PoC showcases the practicality and feasibility of integrating properly configured off-the-shelf hardware, software components and standardized communication technologies to support real-time road-event detection and dissemination.

The experimental results confirm that commercial 5G networks are generally unable to satisfy stringent end-to-end latency requirements below 100 ms for safety-critical vehicular applications. Conversely, the adopted private 5G deployment achieves dissemination delays in the order of 20 ms. When combined with the inference latency contribution, the overall end-to-end delay remains approximately around 80 ms, thus meeting typical latency requirements for safety-related services.

These findings suggest that V2N2X communications for safety-critical applications may require dedicated network slicing mechanisms capable of prioritizing vehicular traffic flows. Nevertheless, the results also indicate that even private 5G deployments may experience latency degradation when the edge server hosting the MQTT broker operates under heavy-load conditions.

Future work will therefore investigate alternative application-layer messaging protocols, such as Advanced Message Queuing Protocol (AMQP), together with optimized broker placement and load-balancing strategies across edge infrastructures.

Moreover, the presence of VRUs on the road will be further considered as a different hazard event to be detected and notified.

## Figures and Tables

**Table 1:** Mapping from detector confidence score (*s*) to DENM informati nQuality.

Confidence Score *s*_*i*_	InformationQuality
0.6≤s<0.65	3
0.65≤s<0.7	4
0.7≤s<0.8	5
0.8≤s<0.9	6
s≥0.9	7

**Table 2:** DENM-related classes detection performance on the validation set.

Group	Precision	Recall	${\mathrm{mAP}}_{50}$	mAP_50–95_
MTSD + city-specific augmentation	0.723	0.529	0.667	0.576
MTSD only	0.537	0.439	0.467	0.386

**Table 3:** Statistics of 5G-related DENM dissemination delay under different 5G network and broker load conditions.

Scenario	Mean [ms]	Min [ms]	Max [ms]	Std. dev. [ms]	95% CI [ms]
5G Private, Edge broker, Light load	19.28	6.00	69.00	7.55	[18.81,19.75]
5G Private, Edge broker, Medium load	37.73	8.00	208.00	21.96	[36.37,39.09]
5G Private, Edge broker, Heavy load	141.67	30.00	447.00	56.91	[138.15,145.20]
5G Public, Remote broker	245.71	89.00	438.00	59.01	[242.05,249.36]

**Table 4:** Duplicate events suppression.

Configuration	Detected Events	Transmitted DENMS	Suppression Ratio
w/o anti-duplication	1000	1000	–
w anti-duplication	1000	75	92.5%

## Data Availability

Data will be provided upon request.

## Abbreviations

The following abbreviations are used in this manuscript:

5G	Fifth Generation	ML	Machine Learning
5GAA	Fifth Generation Automotive Association	MP	megapixel
ADAS	Advanced Driver-Assistance Systems	MTSD	Mapillary Traffic Sign Dataset
AI	Artificial Intelligence	MQTT	Message Queuing Telemetry Transport
AMQP	Advanced Message Queuing Protocol	NLOS	Non-Line-of-Sight
API	application programming interface	NMS	Non-Maximum Suppression
CAM	Cooperative Awareness Message	NR	New Radio
COCO	Microsoft Common Objects in Context	OBU	On Board Unit
CPU	Central Processing Unit	OD	Object Detection
CVAT	Computer Vision Annotation Tool	ONNX	Open Neural Network Exchange
DENM	Decentralized Environment Notification Message	OS	Operating System
ETSI	European Telecommunications Standards Institute	PDU	Protocol Data Unit
FP16	Half-Precision Floating Point	PoC	Proof-of-Concept
FP32	Single-precision Floating Point	RAN	Radio Access Network
FPS	Frame per Second	RSU	Road Side Unit
gNB	gNodeB	S-LDM	Server-Local Dynamic Map
GNSS	Global Navigation Satellite System	SDK	Software Developmet Kit
GPU	Graphics Processing Unit	SIM	Subscriber Identity Module
HD	High Definition	TPC	Texture Processing Cluster
HTTP	HyperText Transfer Protocol	TRT	TensorRT
IoU	Intersection over Union	UE	User Equipment
ISD	Information Sharing Domain	UPF	User Plane Function
ITS	Intelligent Transport Systems	USB	Universal Serial Bus
JSON	JavaScript Object Notation	V2I	Vehicle-to-Infrastructure
LDM	Local Dynamic Map	V2N2X	Vehicle-to-Network-to-Everything
LTE	Long Term Evolution	V2V	Vehicle-to-Vehicle
LTS	aclLTS	V2X	Vehicle-to-Everything
mAP	mean Average Precision	venv	virtual environment
MJPEG	Motion Joint Photographic Experts Group	YOLO	You Only Look Once
